# Characterization of 250 MeV Protons from the Varian ProBeam PBS System for FLASH Radiation Therapy

**DOI:** 10.14338/IJPT-22-00027.1

**Published:** 2023-03-03

**Authors:** Serdar Charyyev, Chih-Wei Chang, Mingyao Zhu, Liyong Lin, Katja Langen, Anees Dhabaan

**Affiliations:** 1 Department of Radiation Oncology, Stanford University, Palo Alto, CA, USA; 2 Department of Radiation Oncology and Winship Cancer Institute, Emory University, Atlanta, GA, USA

**Keywords:** FLASH, proton therapy, commissioning

## Abstract

Shoot-through proton FLASH radiation therapy has been proposed where the highest energy is extracted from a cyclotron to maximize the dose rate (DR). Although our proton pencil beam scanning system can deliver 250 MeV (the highest energy), this energy is not used clinically, and as such, 250 MeV has yet to be characterized during clinical commissioning. We aim to characterize the 250-MeV proton beam from the Varian ProBeam system for FLASH and assess the usability of the clinical monitoring ionization chamber (MIC) for FLASH use.

We measured the following data for beam commissioning: integral depth dose curve, spot sigma, and absolute dose. To evaluate the MIC, we measured output as a function of beam current. To characterize a 250 MeV FLASH beam, we measured (1) the central axis DR as a function of current and spot spacing and arrangement, (2) for a fixed spot spacing, the maximum field size that achieves FLASH DR (ie, > 40 Gy/s), and (3) DR reproducibility. All FLASH DR measurements were performed using an ion chamber for the absolute dose, and irradiation times were obtained from log files. We verified dose measurements using EBT-XD films and irradiation times using a fast, pixelated spectral detector.

R90 and R80 from integral depth dose were 37.58 and 37.69 cm, and spot sigma at the isocenter were σ_x_ = 3.336 and σ_y_ = 3.332 mm, respectively. The absolute dose output was measured as 0.343 Gy*mm^2^/MU for the commissioning conditions. Output was stable for beam currents up to 15 nA and gradually increased to 12-fold for 115 nA. Dose and DR depended on beam current, spot spacing, and arrangement and could be reproduced with 6.4% and 4.2% variations, respectively.

Although FLASH was achieved and the largest field size that delivers FLASH DR was determined as 35 × 35 mm^2^, the current MIC has DR dependence, and users should measure dose and DR independently each time for their FLASH applications.

## Introduction

A sequence of research [1–4] has shown that ultra-high dose rate (DR) (> 40 Gy/s, FLASH) has a sparing effect on normal tissue due to the “FLASH effect.” The seminal work by Favaudon et al [1] demonstrated normal tissue sparing in nude mice, using low-energy electrons to irradiate their target. The same FLASH irradiator was used to treat the first patient with cutaneous lymphoma using FLASH radiation therapy (RT) [5]. Even though they are suitable for skin cancer, electrons are not penetrative enough to produce a useful beam for deep-seated tumors.

Investigations have shown the FLASH effect with x-rays in the kilovolt range [6–9] and efforts to achieve the FLASH effect with megavoltage x-rays [6, 10]. Regardless, an attractive and readily available option to deliver a FLASH beam is cyclotron-based protons, specifically pencil beam scanning (PBS), because PBS can be better controlled in terms of position and intensity [11]. These systems deliver proton therapy at a clinical DR of approximately 1 to 2 Gy/min. Switching to FLASH mode necessitates increasing the DR by orders of magnitude. This is a challenge for treatment delivery systems, especially at low energies, because the energy degradation process causes beam losses, limiting beam currents that reach the treatment rooms.

Recently, techniques for proton beams shooting through the patient from different angles and irradiating the tumor with the plateau region, namely “shoot-through” proton FLASH RT, have been proposed [12, 13]. Even though this means that the tissue at the distal end of the tumor will receive a higher dose relative to conventional proton RT, the proposal of shoot-through FLASH emerged out of an interest in maximizing the DR. To deliver the proton beam at FLASH DR on a cyclotron-based proton therapy system, the inefficient beam degrader needs to be disabled, such that the beam from the cyclotron can be used directly [14].

Even though our PBS system, Varian ProBeam, can deliver a 250-MeV (the highest energy) proton beam. This energy is not used clinically, and as such, the 250-MeV beam has not been characterized during the clinical commissioning [15].

Ionization chambers are routinely used in RT to monitor absolute dose and are integrated into the delivery and monitoring system of our proton therapy system, and we rely on them to switch off the beam promptly. Therefore, their ability to work at a higher DR needs to be assessed before such systems can be released as “FLASH ready” for treatments.

In this study, we aimed to characterize 250-MeV protons from a Varian ProBeam PBS system (Varian Medical Systems, Palo Alto, CA) for FLASH RT and to characterize the currently installed monitoring ionization chamber (MIC) for FLASH beams. We believe this is an important prerequisite for the safe and reliable delivery of FLASH with PBS. By doing this characterization, we can set up a beam model in our treatment planning system and have a set of reference values for subsequent quality assurance for that energy. This information is also essential for Monte Carlo applications, where beam characteristics are needed to set up the phase space parameters and run the simulations to avoid modeling the whole beamline [16].

Currently, few published studies have achieved FLASH DR with 250-MeV protons from Varian's ProBeam system [17–22], but none reported the characteristics of the beam. This is necessary to guide prospective animal studies using the ProBeam system.

## Materials and Methods

### Measurement Data

In the context of this study, we measured the following data needed for beam commissioning as recommended per our treatment planning system, RayStation (version 10B): integral depth dose (IDD) curve, spot sigma, virtual source position, and absolute dose calibration. To test the capabilities of MIC, we measured output as a function of beam current. To characterize 250 MeV for FLASH, we measured (1) the central axis (CAX) DR as a function of current and spot spacing and arrangement, (2) for a fixed spot spacing, the largest field size that still achieves FLASH DR (ie, > 40 Gy/s), and (3) the DR reproducibility. All measurements and conditions are summarized in **Table 1**. Note that 1 nA corresponds to clinical beam current.

**Table 1.** Measurements and conditions performed in this study.

**Quantity**	**Spot map**	**Detector**	**Current (nA)**	**Location**
Spot profile	Single spot	Lynx	1	In air along z-axis at 400, 300, 200, −100, 0, 100 mm. Gantry at 90
IDD	Single spot	StingRay	1	Detector scans in liquid water along CAX. Gantry at 90
Output	10 × 10 cm^2^ square field with 4 mm spot spacing	PPC40	1, 10, 15, 20, 30, 70, 100, 115	In solid water at 2-cm depth. SAD setup. Gantry at 0. Also, with gantry at 90 and SSD setup
DR vs current	35 × 35 mm^2^ square field with 6-mm spot spacing	PPC40 for dose Log files for time	1, 30, 70, 100, 110, 115	In solid water at 10-cm depth. SAD setup. Gantry at 0
DR vs spot spacing	Same # of spots as in (4) but with 4-, 7-, and 8-mm spot spacings	PPC40 for dose Log files for time	115	In solid water at 10-cm depth. SAD setup. Gantry at 0
DR vs spot arrangement	Same # of spots and spacing as in (4) but hexagonal arrangement	PPC40 for dose Log files for time	115	In solid water at 10-cm depth. SAD setup. Gantry at 0
DR vs field size	35 × 35, 30 × 30, and 25 × 30 mm^2^ square field with 6-mm spot spacing	PPC40 for dose Log files for time	115	In solid water at 10-cm depth. SAD setup. Gantry at 0
DR reproducibility	30 × 30 mm^2^ square field with 6-mm spot spacing	PPC40 for dose Log files for time Verified with film and TPX3 for one field	150	In solid water at 10-cm depth. SAD setup. Gantry at 0

**Abbreviations:** IDD, integral depth dose; CAX, central axis; SAD, source-to-axis distance; DR, dose rate.

All measurements with the 250-MeV proton beam were performed in Varian's “commissioning” mode as 250 MeV is available only in commissioning mode. Moreover, the commissioning mode requires a certain file format for delivery/plan files, and they need to be prepared accordingly.

### Spot Sigma Measurements

The spot profiles at 6 locations in the air along the z-axis (400, 300, 200, 100, 0, and −100 mm; the first 4 locations were upstream from the isocenter and the last location was downstream from the isocenter) were acquired for 250-MeV protons using a 2-dimensional scintillation detector Lynx (IBA Dosimetry, Schwarzenbruck, Germany) with 0.5-mm resolution. Spot sigmas in the x direction (σ_x_) and y direction (σ_y_) were determined for each of the 6 locations by fitting the spot profile to a Gaussian distribution. The change in spot profile at different locations from the isocenter along the z axis is used to determine phase space parameters of divergence and correlation coefficients [15] using RayStation 10B [23].

### Integrated Depth Dose Measurements

The IDD curve was measured for a monoenergetic 250-MeV proton beam incident on the CAX of a large-area detector, StingRay (IBA Dosimetry, Schwarzenbruck, Germany), with an active scanning diameter of 12 cm. Integrated charge measurements for the entire Bragg peak were performed with DOSE-1 (IBA Dosimetry, Schwarzenbruck, Germany), a single-channel, high-precision, reference class electrometer by applying a bias of 500 V. The StingRay was scanned in a water tank with microleveling capabilities, Blue Phantom^2^ (IBA Dosimetry, Schwarzenbruck, Germany). It is not easy to use StingRay and water tank with the gantry at 90°; however, because the FLASH beam was initially commissioned and set for use at a gantry angle of 90° at our center, the IDD curve and spot profiles were measured at the gantry angle of 90°. The IDD curve was shifted to account for the water-equivalent thickness of the pin distance, chamber buildup, tank wall thickness, and an additional offset to avoid chamber damage, a total of 13.77 mm. Range measurement from IDD was compared with the continuous slowing down approximation range, as obtained from National Institute of Standards and Technology data for protons [24]. Later, the FLASH was set for use with the gantry at 0°; however, we did not repeat IDD and spot profile measurements at 0° as we did not expect them to change.

### Absolute Dose Calibration Measurements

Absolute field output was measured in solid water, Gammex (Sun Nuclear Corp, Melbourne, Florida), with a 20-mm diameter parallel plate ionization chamber, PPC40 (IBA Dosimetry, Schwarzenbruck, Germany), and DOSE-1 electrometer at 2-cm depth using monoenergetic 10 × 10 cm^2^ square field with 4-mm spot spacing (ie, 26 spots in both x and y directions, with a total of 676 spots). The weight of each spot was 100 MU/spot to create a uniform lateral profile. For commissioning purposes, we measured output with a beam current of 1 nA in a source-to-axis distance setup with a gantry angle of 0°. We then varied the beam current up to 115 nA to see output variations with beam current. We also measured the output at a gantry angle of 90° and with a source-to-surface distance setup to see the dependence of the output on those variables.

### Details of FLASH Measurements

In this study, DR is reported in terms of an average (ie, delivered dose/time of irradiation). DR was measured using PPC40 and DOSE-1, and irradiation times were obtained from the Varian log files. We independently verified the dose measurement using EBT-XD radiochromic film for one of the fields. Verification measurements of the irradiation time were also performed for 1 field using a fast, pixelated spectral detector AdvaPIX-TPX3 (TPX3) [25] placed well beyond the primary beam. DR dependence on beam current measurements was performed using a 35 × 35-mm^2^ field size, with square spot arrangement and a 6-mm spot spacing to deliver 17.5 Gy at a 10-cm depth. All field sizes in this study are geometric field sizes (ie, calculated based on several spots and spot spacings). At the 115-nA beam current, we measured DR dependence on spot spacing using 4-, 6-, 7-, and 8-mm spot spacings and spot arrangement using a hexagonal spot arrangement. In doing this, the number of spots was kept constant. We modified the field size on the fly in commissioning mode to determine the largest field size that still achieves FLASH DR at the beam current of 115 nA. For DR reproducibility, we decreased the field size to 30 × 30 mm^2^ and measured DR repeatedly 10 times at the beam current of 150 nA.

## Results

### Spot Sigma and Integrated Depth Dose

**Figure 1.**
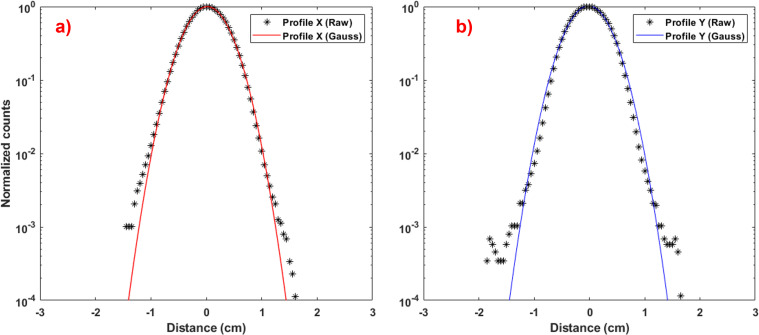
(a) x spot and (b) y spot profiles at isocenter (ie, z = 0) and corresponding fits to the Gaussian distribution, with σ_x_ = 3.336 mm and σ_y_ = 3.332 mm obtained from fitted Gaussian curves (y axis is in log scale).

In **Figure 1**, we present (a) x-spot and (b) y-spot profiles at the isocenter (ie, z = 0) and corresponding fits to the Gaussian distribution, with σ_x_ = 3.336 mm and σ_y_ = 3.332 mm obtained from fitted Gaussian curves. Both x and y profiles fit their Gaussian curve well, with R-square values of 0.9999 and root-mean-square-error values of less than 10^−3^ for both curves.

Spot sigmas, σ_x_, and σ_y_ for all the measured 6 locations are tabulated in **Table 2**. The results in **Table 2** are SD values of the Gaussian distributions obtained by fitting them to the measurement of the spot profiles. They are not direct measurement results. The uncertainty in measurement is dictated by the Lynx measurement resolution of 0.5 mm. From these values, we can determine the phase space parameters of divergence and correlation coefficient as 1.54 mrad and 0.6215. The divergence of the beam shows the angular spread of the beam. For comparison purposes, the angular spreads for the 70-, 150-, and 200-MeV beams from our system are 6.96, 5.24, and 2.62 mrad, respectively. The 250 MeV is the highest (ie, not degraded) energy from our PBS system; hence, we expect the divergence to be even smaller than other energies. The correlation coefficient shows how sigma and divergence are correlated, with unitless positive and negative values indicating a defocusing and focusing beam, respectively. The value reported here aligns well with a defocusing beam because our measurements are at the nozzle exit.

**Table 2.** Spot sigmas, σ_x_, and σ_y_, for all of the measured 6 locations.

**Location (mm)**	**σ_x_ (mm)**	**σ_y_ (mm)**
400	3.084	2.842
300	3.137	3.001
200	3.175	3.101
100	3.229	3.197
0	3.336	3.332
−100	3.388	3.446

R90 and R80 values from IDD curve, as measured with Stingray, were determined as 37.58 and 37.69 cm, respectively.

### Absolute Dose Calibration

**Figure 2.**
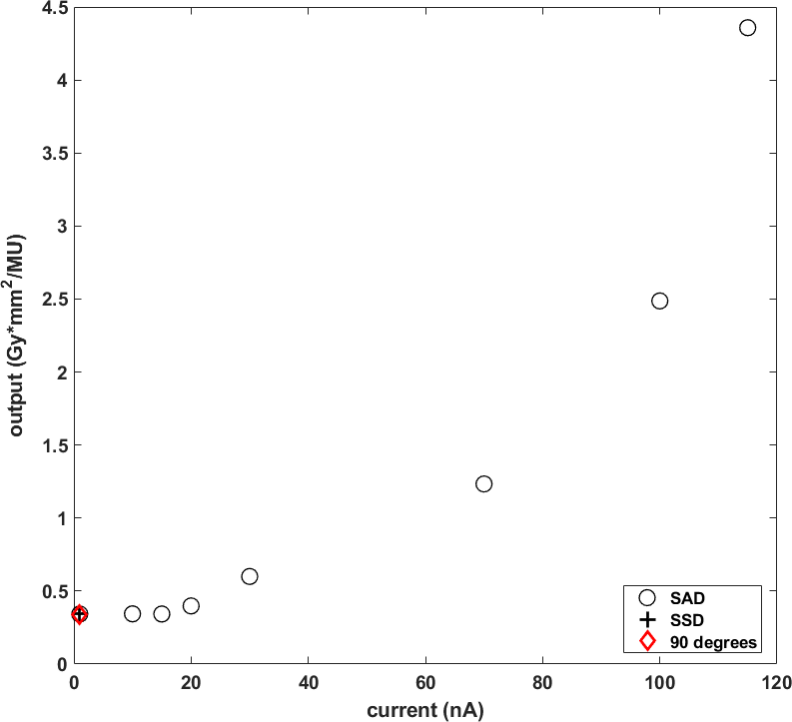
Output as a function of current in SAD setup (shown as black circles), SSD setup (shown as a black plus symbol) and gantry angle (shown as a red diamond symbol). Abbreviations: SAD, source-to-axis distance; SSD, source-to-surface distance.

The absolute dose output was measured as 0.343 Gy*mm^2^/MU for the conditions specified in the Absolute Dose Calibration Measurements section. Output was stable for beam currents up to 15 nA, after which it increased to 4.358 Gy*mm^2^/MU for beam current of 115 nA, as seen in **Figure 2**. Output was measured to be independent of gantry angle (shown as a red diamond symbol in **Figure 2**) and source-to-surface distance/source-to-axis distance setup (shown as a black plus symbol in **Figure 2**) variations.

### FLASH Measurements

**Figure 3.**
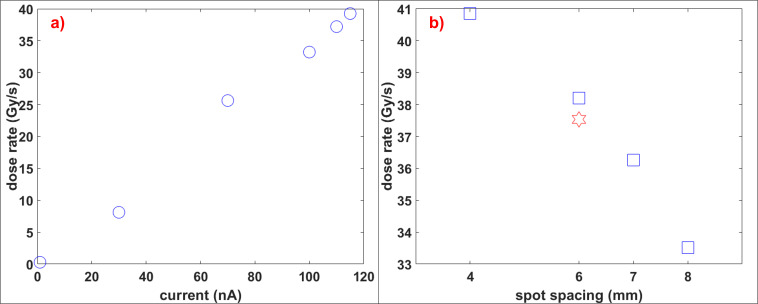
(a) Dose rate as a function of beam current. (b) Dose rate as a function of spot spacing. Red hexagram corresponds to the hexagonal spot arrangement with 6-mm spot spacing.

As seen in **Figure 3**, DR increases with beam current (shown with blue circles in **Figure 3a**) for a 35 × 35 mm^2^ field at a depth of 10 cm with approximately 0.3 Gy/s at 1 nA and approximately 40 Gy/s at 115 nA. Moreover, smaller spot spacings were delivered with a higher DR (shown with blue squares in **Figure 3b**), and hexagonal spot arrangement was delivered with slightly less DR (shown with a red hexagram in **Figure 3b**) than a square arrangement with the same spot spacing. Both increasing the spot spacing and changing it to a hexagonal arrangement decrease the dose slightly, decreasing the DR. For the 115-nA beam current, the largest field size that delivers FLASH DR was determined as 35 × 35 mm^2^.

**Figure 4.**
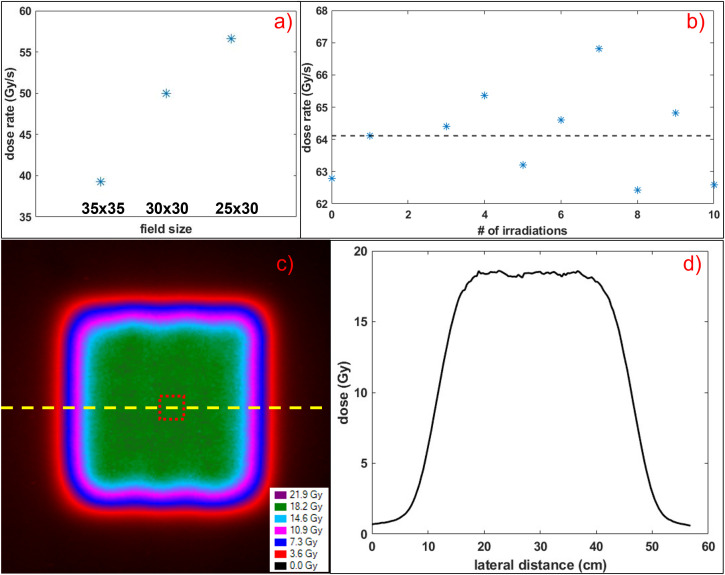
(a) Dose rate as a function of field size at beam current 115 nA. (b) Dose rate reproducibility for a 30 × 30 mm^2^ field delivered at 150 nA beam current for 10 repeated irradiations with an average dose rate of 64.1 Gy/s, shown with a black dashed line. (c) Two-dimensional dose distribution at 10-cm depth in solid water phantom for a 30 × 30 mm^2^ field delivered at 150 nA beam current as measured with EBT-XD. (d) Dose profile through the film as indicated by a yellow dashed line in Figure 4c.

A DR of 50 Gy/s and 56.6 Gy/s can be achieved for 30 × 30 mm^2^ and 25 × 30 mm^2^ field sizes, respectively (**Figure 4a**). DR reproducibility for a 30 × 30 mm^2^ field is illustrated in **Figure 4b**, as delivered with 150 nA beam current. The minimum, maximum, and average DRs were 62.4, 66.8, and 64.1 Gy/s, respectively, and variability of up to 4.2% was observed. For this field, a dose of 17.5 Gy was delivered at a 10-cm depth as measured with PPC40. This was confirmed with EBT-XD measurement, as seen in **Figures 4c** and **4d**, with a dose wash and a profile (yellow dashed line) through the film. The average dose from the region of interest (shown with a red dashed square in **Figure 4c** in the center of the film) was 18.36 Gy, higher than PPC40 measured. The irradiation time for one of the fields was 269 ms from Varian log files and confirmed as 268.9 ms with the TPX3 detector. The fluctuations in DR stem from fluctuations in measured dose. The minimum, maximum, and average measured doses were 16.48, 18.51, and 17.40 Gy, respectively, and variability of up to 6.4% was observed.

## Discussion

Among the results presented in the Results section, several observations are noted and discussed in this section. First, we measured spot profiles and IDD at a 1 nA, non-FLASH beam current. We do not expect the physics (spot size and energy) to change as the DR increases. This has been shown by Darafsheh et al [26], albeit on a different proton system. For the ProBeam system, Langner et al [27] showed no DR dependence by increasing the DR 50-fold. Although they have not reached FLASH DRs, their study shows that beam spot size and energy are not DR-dependent. However, under FLASH conditions, beam optics (due to the space charge effect of densely bunched protons) and beamline configuration (like removal of some component or change of steering parameters) can change, prompting spot profile comparison of conventional and FLASH beams [28, 29]. This type of comparison was not performed in this work and is a limitation of this study. Adhering to vendor DR recommendations with our detectors for IDD (StingRay) and spot size (Lynx), we did not expose them to FLASH DRs. This is out of an abundance of caution, as we expect this will reduce (likely damage) the life of our detectors, especially the StingRay, which must be exposed to the beam for a long time for a low uncertainty IDD curve.

The IDD measurement includes the range uncertainty of ± 1 mm. Because the R80 value minimizes the dependency on initial energy spread [30], the measured R80 is compared with the continuous slowing down approximation range from the National Institute of Standards and Technology. The R80 differences are within 1 and 3 mm when compared with National Institute of Standards and Technology data for 249- and 250-MeV proton ranges, respectively. The result implies that the actual mean energy of the beam from our PBS system is closer to 249 MeV.

In Figure 1, beyond ± 1.5 cm, the raw (measurement) data are 0. This is because the Lynx detector's count range, 0 to 1000, limits its ability to measure low-dose tails with reasonable accuracy; ± 1.5 cm is coincident with counts falling below 10^−3^ (normalized value). This is where the profile deviates significantly from a single Gaussian significantly, as shown previously by several investigators [29, 31, 32]. R-square and root-mean-square-error values are obtained by considering the points above the 10^−3^ threshold. To better characterize the spot profile by including non-Gaussian halo, methods proposed by Lin et al [32] should be followed.

Second, we would like to discuss the performance of the detectors used in this work under high DRs. In our study, PPC40 was used at 150 nA (up to 65 Gy/s). PPC40's performance was investigated extensively before by Yin et al [33] under FLASH conditions, and they have not found significant ion recombination at 63.7 Gy/s. They reported ion recombination correction factor of 1.0009 at 63.7 Gy/s using a 2-voltage method. Using a similar method, we have also measured an ion recombination correction factor as 1.0007 at 65 Gy/s.

With EBT-XD film, we measured a dose 4.9% higher than what PPC40 measured. Villoing et al [34] observed a similar overresponse (up to 4.5%) in dose measurements between the conventional and FLASH DRs using EBT-XD films. In their work, they delivered 20 Gy to the EBT-XD films, which is close to what we have delivered in our study.

TPX3 detector saturates under the primary beam with dose rates used in this study, but because the detector was placed well beyond the primary beam, it received low flux. We measured irradiation times, taking prompt gammas as surrogates for the primary protons beam. We have shown the feasibility of this method using TPX3 in another study, details of which can be found in Charyyev et al [35]. The TPX3 has a time resolution of 1.6 ns [36]. Our other work [35] investigated the timing measurements with this detector on the microsecond scale. We also performed reproducibility of the irradiation times with this detector, where we measured the irradiation time of a single spot 17 times to obtain an average reading of 47.8 ms with SD ± 0.6 ms.

Third, output was found to be stable up to a beam current of 15 nA, after which we have shown DR dependence of the MIC. Thus, the clinical MIC currently installed in ProBeam systems will not shut off the beam at the requested dose. This is likely because of higher ion recombination effects [37], which was demonstrated by Liszka et al [38] and Yin et al [33] to induce significant ion collection issues even at 13 Gy/s DR level. Sørensen et al [19] reported an ion collection efficiency of 7% to 10% of the same system at 215 nA. Ion collection efficiency at 115 nA is 7.8% for our system, as evident from **Figure 2**. Until the design of the MIC is modified to account for this and the subsequent replacement of MIC by the vendor, users should independently measure dose and DR each time for their FLASH applications.

We have observed through reproducibility measurements that the dose and DR can vary by 6.4% and 4.2%, respectively, for repeated irradiations. This warrants caution. Because of the instabilities of the MIC to control the beam, secondary, possibly tertiary, confirmation measurements of the dose and DR must be performed. This uncertainty also needs to be considered when interpreting outcomes of studies with the current clinical system.

For most FLASH studies, DR is reported as an average (ie, delivered dose/time of irradiation), which was the definition we adopted in this study. Even though there is a consensus that average DR is most relevant for the FLASH effect [39–41], for proton PBS, DR can be defined at each point in the field as the sum of contributions from multiple spots [42, 43]. In that sense, considering the subset of voxels in a field, the DR can be higher than the reported DR in this work. It is relevant to study DR in the context of sub-voxels because the processes that are believed to be responsible for the FLASH effect happen at the cellular level.

Finally, the Varian log files record the irradiation time for individual spots. We used the sum of the irradiation time for individual spots in the denominator of the dose rate calculation. However, there is a transition time in going from one spot to another. Kanouta et al [44] measured transition durations as 0.20 ± 0.04 ms (5.1-mm steps) for faster magnets and 0.50 ± 0.04 ms (5.0-mm steps) for slower magnets. Because our spot map is very small and interspot distances are short, the spot transition time (1-2 ms) is short compared with the sum of the irradiation time (270 ms) of the individual spots. This is evident from the log files being very close to the TPX3 measurement. Therefore, the sum of the irradiation times for individual spots from log files in the denominator of the DR calculations is a good approximation in the context of this work.

### Conclusion

In conclusion, we characterized the 250-MeV proton beam and MIC from a Varian ProBeam PBS system for FLASH RT. The values we provided will serve as reference values for quality checks. Moreover, the essential beam phase space parameters are provided to explore FLASH effects using proton Monte Carlo applications. We investigated the clinical MIC for FLASH-readiness and found that the output is DR-dependent, which is not expected from a stable PBS system. We also have shown that delivered dose and DR fluctuates between identical deliveries, in our case, by up to 6% and 4%, respectively, for repeated irradiations. The implications are that users should make independent and repeated measurements of dose and DR until the MIC design is changed to accurately monitor the FLASH beam.
